# Genetics and beyond: Precision Medicine Real-World Data for Patients with Cervical, Vaginal or Vulvar Cancer in a Tertiary Cancer Center

**DOI:** 10.3390/ijms25042345

**Published:** 2024-02-16

**Authors:** Fabian B. T. Kraus, Elena Sultova, Kathrin Heinrich, Andreas Jung, C. Benedikt Westphalen, Christina V. Tauber, Jörg Kumbrink, Martina Rudelius, Frederick Klauschen, Philipp A. Greif, Alexander König, Anca Chelariu-Raicu, Bastian Czogalla, Alexander Burges, Sven Mahner, Rachel Wuerstlein, Fabian Trillsch

**Affiliations:** 1 Department of Obstetrics and Gynecology, Comprehensive Cancer Center Munich, University Hospital, LMU Munich, Marchioninistr. 15, 81377 Munich, Germany; 2 Massachusetts General Hospital, Harvard Medical School, Boston, MA 02114, USA; 3 Department of Medicine III, Comprehensive Cancer Center Munich, University Hospital, LMU Munich, Marchioninistr. 15, 81377 Munich, Germany; 4 Institute of Pathology, Comprehensive Cancer Center Munich, LMU University Hospital, Ludwig Maximilians University (LMU), 81377 Munich, Germany; 5 German Cancer Consortium (DKTK), Partner Site Munich, 81377 Munich, Germany; 6 German Cancer Research Center (DKFZ), 69121 Heidelberg, Germany

**Keywords:** molecular tumor board, precision medicine, human papillomavirus, targeted therapies, antibody–drug conjugates, checkpoint inhibitors, kinase inhibitors, cervical cancer, vaginal cancer, vulvar cancer

## Abstract

Advances in molecular tumor diagnostics have transformed cancer care. However, it remains unclear whether precision oncology has the same impact and transformative nature across all malignancies. We conducted a retrospective analysis of patients with human papillomavirus (HPV)-related gynecologic malignancies who underwent comprehensive molecular profiling and subsequent discussion at the interdisciplinary Molecular Tumor Board (MTB) of the University Hospital, LMU Munich, between 11/2017 and 06/2022. We identified a total cohort of 31 patients diagnosed with cervical (CC), vaginal or vulvar cancer. Twenty-two patients (fraction: 0.71) harbored at least one mutation. Fifteen patients (0.48) had an actionable mutation and fourteen (0.45) received a recommendation for a targeted treatment within the MTB. One CC patient received a biomarker-guided treatment recommended by the MTB and achieved stable disease on the mTOR inhibitor temsirolimus for eight months. Factors leading to non-adherence to MTB recommendations in other patient cases included informed patient refusal, rapid deterioration, stable disease, or use of alternative targeted but biomarker-agnostic treatments such as antibody–drug conjugates or checkpoint inhibitors. Despite a remarkable rate of actionable mutations in HPV-related gynecologic malignancies at our institution, immediate implementation of biomarker-guided targeted treatment recommendations remained low, and access to targeted treatment options after MTB discussion remained a major challenge.

## 1. Introduction

Despite significant advancements in the prevention and treatment of gynecologic malignancies, these cancers are a continuous cause of women’s morbidity and mortality worldwide. Among gynecologic cancers related to human papillomavirus (HPV), cervical cancer (CC) stands as the fourth leading cause of cancer in women globally, with a mortality-to-incidence ratio of 57% according to the GLOBOCAN 2020 data. Following CC, vulvar and vaginal cancers rank twenty-first and twenty-eighth in incidence, with mortality-to-incidence ratios of 38.5% and 44.6%, respectively. For patients in an advanced stage, survival rates decrease dramatically to approximately 5% for CC patients three years after tumor recurrence [1].

Infection with human high-risk HPV types is a major risk factor for the development of CC, along with nicotine use, low socioeconomic status and immunosuppression [2]. Expression of viral oncoproteins, such as E6 and E7, by an infected cervical epithelial cell induces inactivation of the tumor suppressors p53 and retinoblastoma (Rb), leading to unleashed proliferation and accumulation of DNA repair defects [3], such as homologous repair deficiency (HRD) [4] or DNA mismatch repair deficiency (dMMR) [5]. The increasing dedifferentiation of cervical epithelial cells is reflected by the histopathologic diagnosis of cervical intraepithelial neoplasia (CIN) grade 1–3 and ultimately culminates in the development of invasive CC [6]. In addition, HPV was found to play an analogous role in the development of (pre)cancerous vulvar (vulvar intraepithelial neoplasia usual type, uVIN) [7] and vaginal lesions (vaginal intraepithelial neoplasia, VAIN) [8,9].

Traditionally, first-line therapy for CC with curative intent primarily focuses on unimodal management, either radical surgical treatment or platinum-based chemoradiation [10]. In vulvar and vaginal cancer or in specific risk constellations, a combination of both modalities can be applied depending on intraoperative findings and pathology. In addition, current research hints at a potential role for treatment approaches combining immune checkpoint inhibitors with immunostimulators or HPV vaccines [11]. 

Advances in molecular tumor profiling and biomarker testing have generated a wealth of data that have led to a better understanding of oncogenesis and the development of highly specific “targeted therapies” [12,13], the introduction of which has also changed the treatment landscape for gynecologic cancers.

In CC, several targeted but biomarker-agnostic approaches could be successfully integrated into the systemic treatment of recurrent disease with different approvals over the past years. Among these, first, the anti-angiogenic vascular endothelial growth factor (VEGF) inhibitor bevacizumab improved progression-free survival (PFS) and overall survival (OS) for advanced CC patients as a combination partner for platinum-based chemotherapy and as subsequent maintenance treatment [14]. 

Second, pembrolizumab, an anti-PD-1 checkpoint inhibitor (CPI), in combination with platinum-based chemotherapy, improved overall response rate, PFS and OS of patients with metastatic CC [15]. This is consistent with the clinical observation of CC as an immune-related malignancy with a higher prevalence in immunocompromised patient cohorts [16]. It is also in line with the close relationship between CC oncogenesis and DNA repair defects (dMMR, HRD) leading to the emergence of potentially immunogenic tumor neoantigens [17]. In this context, the ESMO Next Generation Sequencing (NGS) Recommendations had advocated testing of tumor mutational burden (TMB) in cervical cancer patients as early as 2020 [18], given the clinical benefits of checkpoint blockade in patients with dMMR deficiency or high microsatellite instability (MSI-H) observed in the KEYNOTE-158 trial. Considering these clinical successes, pembrolizumab was finally approved by the U.S. Food and Drug Administration (FDA) for PD-L1-positive CC in 2022. Interestingly, however, in 2023 a combinatorial approach of checkpoint blockade and VEGF inhibition was shown to mediate a PFS and OS benefit even independently of PD-L1 expression levels [19]. 

Third, and most recently, the activity of tisotumab–vedotin (TV-1), an antibody drug conjugate (ADC) targeting tissue factor (TF), with a promising overall response rate of 24% in metastatic CC patients who had already progressed on or after chemotherapy [20], was confirmed in the phase III ENGOT-cx12 trial, which showed an improvement in OS with TV-1 compared to investigators’ choice chemotherapy [21].

Given these recent therapeutic innovations, the need for biomarker-guided patient selection to maximize treatment efficiency and minimize side effects has become increasingly evident and is subject to ongoing research [22]. In contrast to the aforementioned biomarker-agnostic targeted therapies (CPIs, anti-VEGF inhibitors and ADCs), biomarker-guided treatment algorithms are scarce in the standard of care for cervical, vulvar and vaginal cancer patients, and the integration of molecular diagnostics and result interpretation into clinical practice remains challenging. In this study, we present real-world evidence on the clinical relevance of molecular tumor board (MTB) recommendations for the therapeutic decisions and clinical outcomes of HPV-related gynecologic malignancies at the Department of Obstetrics and Gynecology, University Hospital, LMU Munich, a leading expert center for gynecologic oncology in Germany.

## 2. Results

### 2.1. Patient Cohort

**Figure 1:**
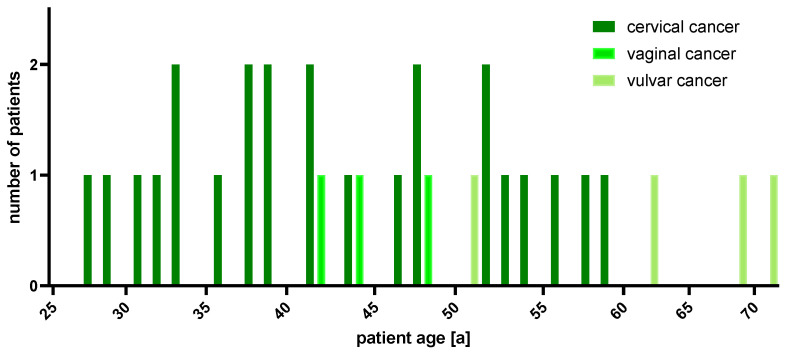
Age distribution at first diagnosis.

A total of 31 female patients with HPV-related cervical, vaginal or vulvar cancer who were discussed at the MTB of the Department of Obstetrics and Gynecology, University Hospital, LMU Munich, Germany, between November 2017 and June 2022, were included in the present patient cohort. Median patient age was 45.7 years (range: 28 to 71 years) at first diagnosis and 48.2 years (range: 28 to 72 years) at the time of MTB presentation (Figure 1). Tissue samples used for genetic diagnostics were collected between 12/2016 and 06/2022.

**Figure 2:**
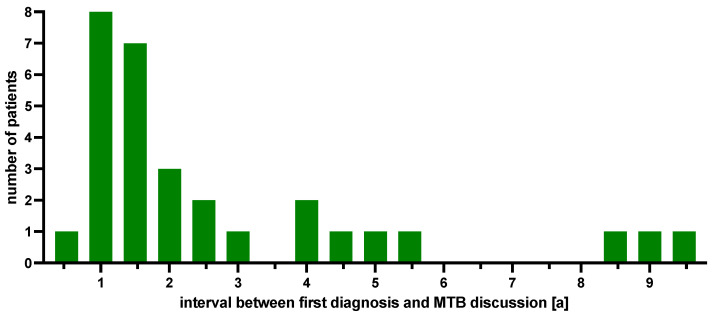
Patient numbers according to the interval between first diagnosis and MTB presentation.

At the time of MTB presentation, all patients were in an advanced tumor stage (i.e., 3 patients in FIGO stage IIIB, one in FIGO stage IIIC and 27 patients in FIGO stage IVB), with a median interval of 2.5 years (range: 0.2 to 9.5 years) from their initial diagnosis (Figure 2). 

**Figure 3:**
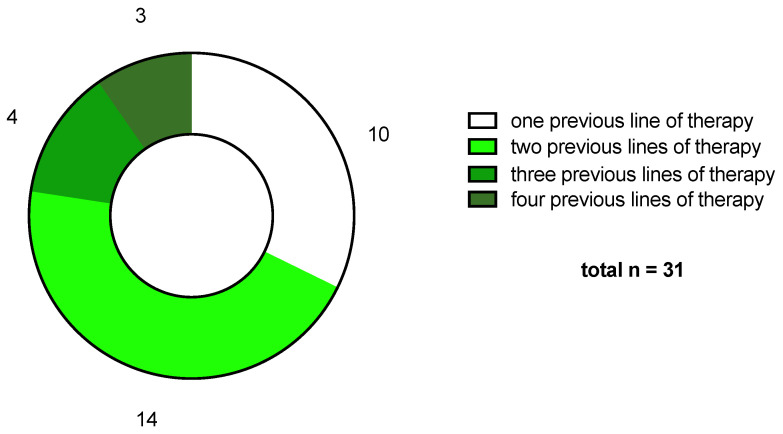
Patient characteristics: previous lines of therapy.

All patients had undergone a median of two lines of therapy (range: one to four) (Figure 3) and had a median number of two metastatic sites (range: zero to five). Metastases were mostly located in lymph nodes (fraction: 0.39), followed by soft tissue (0.18), lungs (0.17), peritoneum (0.14), liver (0.07), kidney (0.04) and brain (0.02).

A total of 24 of the 31 patients (0.77) included in this study were diagnosed with CC, 4 with vulvar cancer (0.13) and 3 patients with vaginal cancer (0.10).

**Figure 4:**
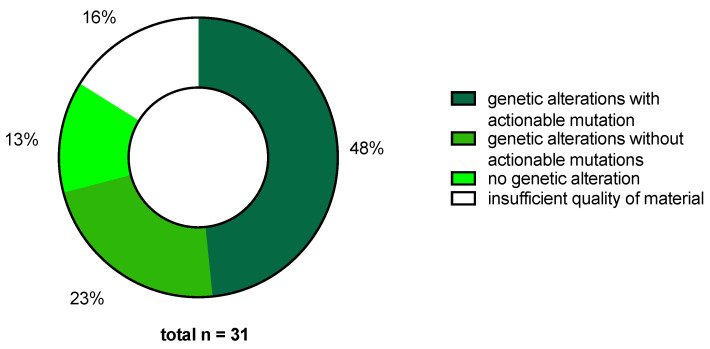
Results of molecular profiling.

In 22 of the 31 patients (0.71), at least one mutation was identified. In 17 cases (0.55), multiple mutations were detected. Among the 22 patients with at least one mutation, actionable molecular alterations were identified in 15 cases (0.68, 10 CC, 3 vulvar cancer, 2 vaginal cancer). Of the described 75 mutations, 18 (0.24) were considered actionable and 57 non-actionable (0.76). No molecular alteration was detected in the tumor specimens of 9 patients (0.29). Tumor sequencing was repeated in 4 cases (0.13) due to insufficient quantity or quality of tissue. Finally, in 5 of the 31 cases (0.16), genomic sequencing failed to retrieve any results (Figure 4).

**Figure 5:**
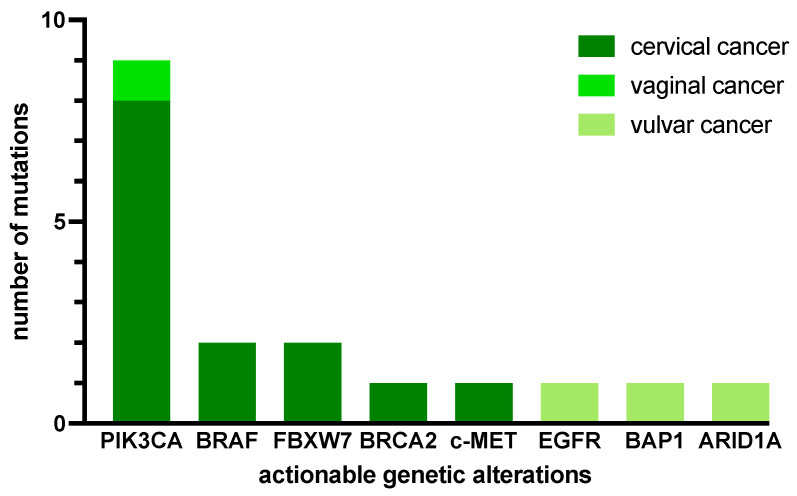
Frequency of actionable genetic alterations per tumor entity.

NGS analysis revealed a total of 75 mutations in 35 different genes in the tumor samples of 22 patients, with a median of 2 molecular alterations per patient (range: 0 to 8). Most alterations affected the TP53 gene (9/75; 0.12), the PIK3CA gene (9/75; 0.12) and the KRAS gene (6/75; 0.08). Among the 15 cases with actionable mutations, PIK3CA alterations were seen in 9 patients (0.50), and BRAF and FBXW7 mutations in 2 patients (0.11), respectively (Figure 5). 

### 2.2. MTB Recommendations

For 18 of 31 patients (0.58), the MTB discussions resulted in 26 recommendations. These recommendations were fifteen specific treatment recommendations with targeted therapies (0.58) and three trial recommendations (0.12). 

Most frequently, the PIK3CA-AKT-mTOR pathway was identified as targetable in 8 of the 15 patients with drug recommendations (0.53). Among these cases, specific biomarker-guided recommendations included an mTOR inhibitor in 5 patients and a PIK3CA inhibitor in 3 cases, both due to a mutation in either the PIK3CA gene or the FBXW7 gene.

**Figure 6:**
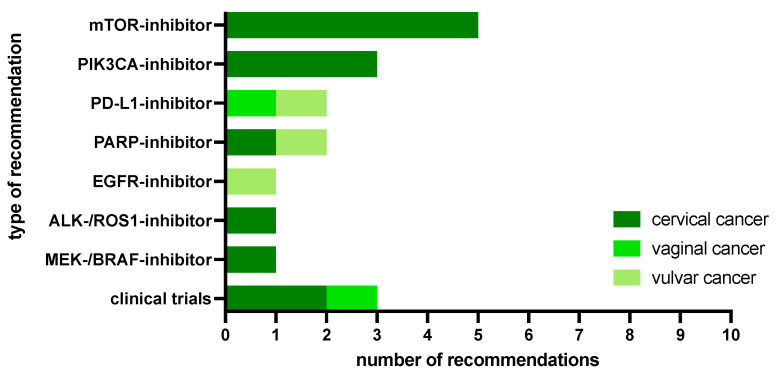
MTB recommendations.

Focusing on other pathways, the following therapeutics were recommended: PARP inhibitors in 2 cases (due to one BRCA2 mutation in a CC patient and a BAP-1 mutation in a vulvar carcinoma patient), a PD-L1 inhibitor in 2 patients (due to one ARID1A mutation in a vulvar cancer and a high TMB in a vaginal cancer patient), a MEK/BRAF inhibitor in one CC patient (due to a BRAF mutation), an ALK/ROS1 inhibitor in one CC patient (due to a c-MET exon 14 skipping mutation) and an EGFR inhibitor in one vulvar cancer patient (due to an EGFR amplification). One CC patient received more than one treatment recommendation with everolimus and crizotinib due to an activating PIK3CA and a concurrent c-MET exon 14 skipping mutation (Figure 6). Table S1 provides a comprehensive list of all patient cases with druggable molecular alterations along with the corresponding therapeutic recommendations made by the MTB.

Overall, targeted therapies were suggested in 14 of 31 patients (0.45), with 10 of 24 CC patients (42%), 3 of 4 vulvar cancer patients (0.75) and one of 3 vaginal cancer patients (0.33). Despite actionable KRAS mutations, 4 CC patients were not recommended a corresponding targeted drug at the respective MTBs from 2018 to 2020, as sotorasib, the first clinically approved KRAS antagonist, was granted FDA approval in 2021. Moreover, 3 patients were suggested inclusion in an active clinical trial, i.e., the National Center for Tumor Diseases (NCT) Master Program for 2 CC patients and the Bavarian Center for Cancer Research (BZKF) Early Clinical Trial Unit (ECTU) Board for one vaginal cancer patient.

In contrast, 17 of the 31 patients (0.55), did not receive any treatment recommendation from the MTB. The main reasons included the absence of tumorigenic mutations (4/31, 0.13), the presence of non-actionable mutations (7/31, 0.23), unsuccessful sequencing due to insufficient tissue quality (5/31, 0.16) and the death of one patient prior to MTB discussion (1/31, 0.03).

### 2.3. Clinical Implementation of MTB Drug Recommendations

Despite the treatment recommendations for targeted therapies in 14 patients, only one MTB drug recommendation was clinically fully implemented (0.07). This case, a CC patient initially diagnosed with an already advanced stage, received upfront chemoradiation and, following the first relapse three months later, palliative combination chemotherapy with cisplatin, paclitaxel and bevacizumab. After a 6-month period of stable disease, another progression was noted. In accordance with the MTB recommendation, the mTOR inhibitor temsirolimus was administered as post-last-line therapy following cost approval of the insurance company. Disease control was achieved for a period of eight months with good tolerability confirmed by the patient. 

**Figure 7:**
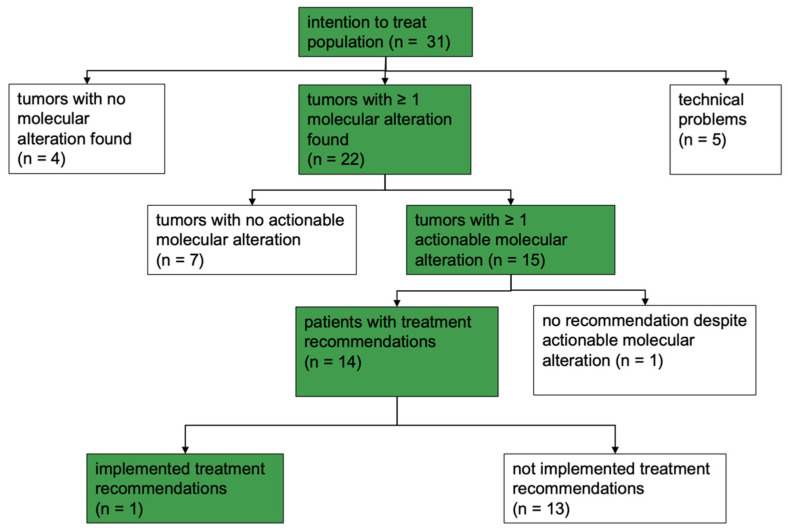
MTB output overview; n: number of patients.

In contrast, in 13 of the 14 patients (0.93), the biomarker-guided therapeutic suggestions from the MTB were not clinically implemented (Figure 7). 

**Figure 8:**
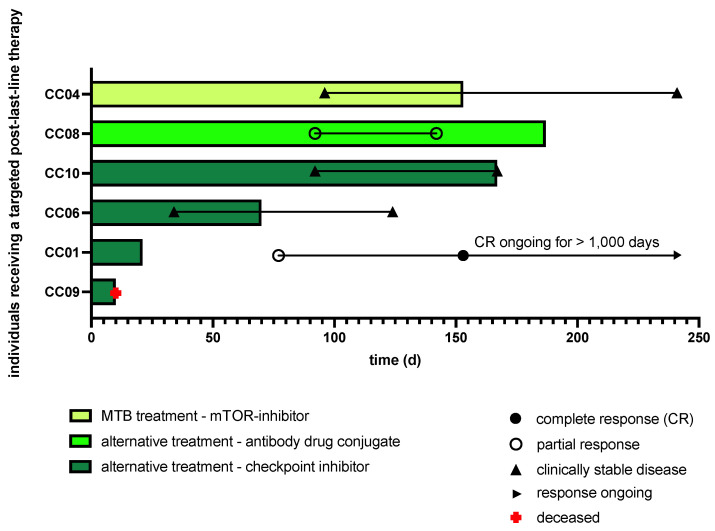
Swimmer plot illustrating the individual clinical course of the 6 patients who either received the biomarker-guided targeted therapy recommended by the MTB (mTOR inhibitor temsirolimus) or a biomarker-agnostic targeted therapy (i.e., an antibody–drug conjugate or a checkpoint inhibitor). Patient identifiers refer to Table S1.

The main reasons for not following the recommendations were that alternative targeted but biomarker-agnostic drugs with published clinical evidence were available, which, in some cases, had been approved for other cancer entities or in a histopathology-agnostic setting in Germany. In the 5 patients with CC, the gTB opted for CPIs (4 cases) or the FDA-approved ADC tisotumab–vedotin (one case) as biomarker-agnostic treatment options (Figure 8).

**Figure 9:**
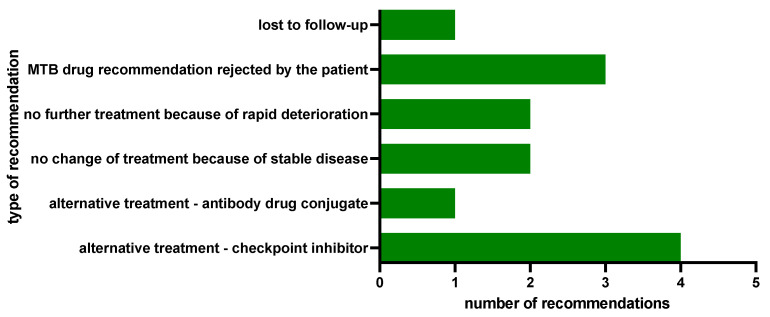
Reasons for non-implementation of MTB recommendations and alternatively implemented therapies by the gTB.

In another 4 patients, the MTB recommendation was not followed by the gTB due to the patients’ clinical courses, as two of them had achieved a stable disease under their previous therapies until data cut-off. Two further patients, in contrast, experienced a rapid clinical deterioration rendering initiation of the therapy suggested by the MTB impossible, favoring palliative care. Another 3 patients rejected the MTB treatment recommendation, and one patient was lost to follow-up (Figure 9). 

## 3. Discussion

Over the past years, advancements in genetic sequencing have revolutionized clinical diagnostics by introducing new, more rapid and cost-effective testing methods. This established the new field of precision medicine, a clinical approach with biomarker-guided and -agnostic targeted therapeutics as an alternative or complement to conventional therapies. However, there is ongoing debate as to which extent NGS can detect druggable targets which can be effectively addressed across all cancer types, regardless of their tissue of origin. The first successful example of this approach in gynecologic oncology was the approval of pembrolizumab for dMMR patients across all cancer types with high efficacy, especially in endometrial cancer patients as more than 20% of them harbor genetic features characteristic for dMMR [23]. 

In accordance, most clinical trials conducted so far have focused on histology-agnostic patient cohorts across many different cancer types, providing a broad overview but limiting understanding of the specific benefits of precision medicine for defined tumor subgroups, especially in rare diseases. For example, a global meta-analysis of 32,149 patients with a wide range of cancers demonstrated clinical benefits for patients who received personalized treatments compared to standard of care [24]. These benefits included a higher median response rate (0.31 vs. 0.10), longer median PFS (5.9 vs. 2.7 months), and improved OS (13.7 vs. 8.9 months) [25]. Although further studies involving different cancer types supported these findings, other clinical studies failed to prove the superiority of the precision medicine approach [25,26,27]. 

In contrast, tumor-subtype-specific real-world evidence on the benefits of precision medicine is weak, especially for infrequent gynecologic malignancies, such as HPV-related malignancies of the female reproductive system [28,29,30]. Accordingly, the present study analyzed the clinical course of 31 patients with HPV-related gynecologic malignancies who were evaluated at the MTB of the University Hospital, LMU Munich between 2017 and 2022. Of these patients, a total of 26 (0.84) underwent successful genetic testing, which ultimately resulted in an MTB statement. The overall size of this cohort (n = 31) is in accordance with analyses published by other cancer centers, e.g., the Medical University of Vienna, Austria [31], the Hospital Universitario Fundación Jiménez Díaz [32] or the University of Oklahoma Health Sciences Center, USA [29]. 

The proportion of gynecologic patients with cervical, vaginal or vulvar cancer testing positive for at least one molecular alteration in our study (0.71) is modestly lower than the proportion (0.90) reported by Taghizadeh et al. 2020, who analyzed 72 gynecologic cancer patient cases at the Medical University of Vienna. This might be due to a different composition of their patient cohort, which included forty-four ovarian but only two cervical and two vaginal cancer patients [31].

In addition, the rate of 0.48 HPV-related cancer patients whose tumors had an actionable mutation was similar to the 0.53 in the LMU breast cancer cohort. Among the first 1000 patients of any cancer type who were discussed at the MTB of our comprehensive cancer center, however, the rate of patients with targetable genomic alterations was slightly lower, at 0.41 [33], a number which is consistent, for example, with the proportion of targetable genomic alterations (0.38) found in the National Cancer Institute Molecular Analysis for Therapy Choice (NCI-MATCH) cohort [34] or in a cohort of 299 gynecologic cancer patients at the University of Buffalo (0.33) [35]. These rates also align with retrospective findings reported by Tannock et al., indicating that “out of every 1000 patients, approximately 400 will exhibit a targetable mutation, and around 120 will be prescribed a corresponding medication” [36]. 

The identification of targetable mutations and the subsequent treatment recommendation by an interdisciplinary expert panel as part of a structured program increases the likelihood of reimbursement by a patient’s health insurance company and thus paves the way for clinical implementation. For this reason, patients were usually referred to the MTB after the last line of standard therapy had been administered, but before all possible therapeutic options had been exhausted, to anticipate the time pressure that usually arises when the patient’s health deteriorates rapidly, and reimbursement must still be negotiated with insurance companies. As a result, the gTBs that followed MTB discussions had to weigh different therapeutic alternatives, resulting in the immediate clinical implementation of a biomarker-guided MTB treatment recommendation in only one of fourteen patient cases. Understanding this implementation rate requires careful consideration of the precise timing of the MTB within the patient’s clinical course and may at least partly be explained by the different types of treatments that gTBs and MTBs tend to focus on, i.e., biomarker-agnostic and biomarker-guided therapies, respectively.

At the time of the MTB discussions, alternative but biomarker-agnostic therapies with published clinical evidence for patients with HPV-related cancers and/or approval in Germany were available. Consequently, biomarker-guided treatment regimens, as suggested by the MTB, could be reserved for later consideration. Instead of following the biomarker-based recommendation of an mTOR, PARP or PIK3CA inhibitor, our gTB therefore leaned toward biomarker-agnostic drugs at this point. In four out of fourteen cases, a checkpoint inhibitor was prescribed which previously had been demonstrated to be effective regardless of PD-L1 expression status [19,37]. In another case, the gTB favored the recently FDA-approved ADC tisotumab–vedotin over a PIK3CA inhibitor, which, as shown in the ENGOT-cx6 trial, can be used regardless of TF expression status [20].

The promising clinical data on the potential of biomarker-agnostic drugs, such as CPIs [24] and ADCs [20], significantly increased the likelihood of reimbursement by health insurance companies. 

In contrast, most evidence for biomarker-guided targeted drugs against cervical, vaginal and vulvar cancer, such as mTOR/PIK3CA and PARP inhibitor-based therapies, stemmed from preclinical data, as most of the drugs suggested by the MTBs had only sporadically been tested in clinical settings for these diseases. For instance, dactolisib, a dual anti-PIK3CA/mTOR inhibitor, exhibited promising antitumor efficacy in an in vitro model for vulvar cancer by counteracting epithelial-to-mesenchymal transition [38]. Additionally, the PHOENIX phase I trial suggested a combination of platinum-based radiochemotherapy with daily everolimus administration, confirming its tolerability but lacking a definitive statement on therapeutic efficacy [39]. 

Similarly, PARP inhibitors, known for their clinical activity in ovarian cancer patients, have mostly been studied in preclinical models in the context of HPV-related malignancies. Promising clinical results on PARP inhibitor efficacy have mainly been published in case reports or early clinical trials [40,41]. In the later clinical course, however, those biomarker-guided targeted therapies might constitute valuable treatment perspectives, underscoring the influence of MTBs, especially in advanced lines of therapy. 

This observation might imply a rationale for postponing genetic profiling until even later in a patient’s clinical course. Yet, upon revisiting our patient cohort, concerns arise regarding further delaying molecular diagnostics. In over a third of cases featuring actionable genomic alterations, our gTB refrained from implementing the medication recommended by the MTB, due to either a rapid deterioration in the patients’ conditions (two out of fourteen cases) or the patients’ refusal of the suggested therapy (three out of fourteen cases). In another case, a patient died after the NGS was completed but before her case could be presented to the MTB. This, on the contrary, argues in favor of holding the MTB earlier in a patient’s clinical course to preserve the information for later treatment decisions, but also illustrates the complexity of determining the optimal time for molecular testing.

On the one hand, tissue specimens for molecular diagnostics should be obtained as late as possible to ensure the genetic representativeness of the tumor in its current state. This is particularly important as intratumor heterogeneity and clonal evolution under selective therapeutic pressure result in both the acquisition of drug resistance as well as the emergence of new therapeutic targets [42]. For instance, in 2015, Tan et al. compared the mutational profiles of breast cancer patients before and after only one cycle of doxorubicin- or docetaxel-based chemotherapy and found new mutations being introduced in 20% of tumors which had initially been classified as wildtype [43]. Not only alterations in tumor mutational profiles [44,45] but also treatment-induced changes in the tumor transcriptome [46,47] and the tumor microenvironment [48,49] were shown to be associated with acquired resistance to both chemotherapy and targeted drugs. The (intratumoral) heterogeneity of molecular biomarkers, however, also underscores the complexity of finding the right therapeutic target. Since an estimated five to ten driver mutations are required for malignant transformation of a previously healthy cell, but a plethora of passenger mutations can occur (e.g., >100,000 mutations of presumed limited or no biological consequence in hypermutated endometrial cancer), extensive research is required to define the most potent therapeutic targets and appropriate biomarkers for their identification [50]. In addition, as different subclonal cancer cell populations will respond differently to targeted therapies depending on their biomarker profile, future research must focus on the optimal therapeutic sequence of targeted drugs. 

On the other hand, patients in advanced tumor stages often experience rapid deterioration, which was identified as one of the main reasons why recommended therapies were not received, both in the literature and our study population [28,33,51]. In addition, the median turnaround time of 19 days (range: 8 to 44) between indication for molecular testing and receipt of NGS results, coupled with the time required for insurance reimbursement approval, may exacerbate the situation and be suboptimally long, particularly for patients with advanced tumors who require rapid interventions to achieve remission [52].

Nevertheless, the implementation of MTBs in clinical practice offers an additional advantage by serving as an educational platform for clinicians in the field of molecular oncology. MTBs raise awareness, deepen understanding and improve confidence among clinicians about current trends and developments in molecular therapeutics [53]. In addition, they help address treatment disparities among patients of different socioeconomic backgrounds [54]. However, as the experience with MTBs grows, there is a need for greater standardization in terms of logistic structure and integration into clinical cancer care. The lack of mandatory guidelines and quality criteria for cancer centers may contribute to the controversial results observed in clinical trials evaluating the effectiveness of precision oncology approaches [51,52]. Variations among cancer centers exist in terms of patient selection criteria, the choice of multigene panels for molecular profiling, criteria for defining mutation targetability, access to off-label drugs and clinical trials and quality control measures [51]. 

As precision medicine workflows continue to improve and the use of biomarkers to define new therapeutic strategies in the second line and beyond has already been proposed for several malignancies [55], consideration should be given to integrating these biomarkers into the first-line setting after validation in large prospective cohorts. Through such integration, a more refined personalization of treatment approaches can be achieved, potentially optimizing drug tolerability and anticancer efficacy from the outset of diagnosis.

In addition, the increased utilization of liquid biopsies promises to save time and provide a more representative analysis in cases of disseminated malignancies. However, there are still ongoing debates regarding the sensitivity of liquid biopsies, and cost considerations remain significant [56,57]. In our analysis, we did not utilize liquid biopsies for molecular-genetic analysis due to concerns about false negative results and the high costs associated with employing highly sensitive systems, especially given the low likelihood of health insurance reimbursement. In the future, the use of liquid biopsy in addition to tumor analysis will allow us to develop even more effective tests in terms of sensitivity and specificity, especially for patients with late-stage disease. Its reduced invasiveness compared to biopsy may lower the threshold for late-stage patients to consent to repeat sampling, thereby improving the timing of genetic testing and the representativeness of its results. 

Although our study provides valuable insights into real-world evidence on molecular diagnostics and targeted therapies in patients with gynecologic malignancies, it is important to acknowledge its limitations. First, the inclusion of a highly selected patient cohort resulted in a relatively small sample size of 31 patients in advanced tumor stages who had already undergone multiple previous therapies [28,29,52]. Thus, fewer unadministered treatment options were left, which potentially limits the generalizability of our results beyond highly selected (post-)last-line therapy patient cohorts. Second, the definition of targetable mutations is complex and requires standardized, widely accepted and regularly updated criteria. Third, it is important to consider the temporal aspect of tumor evolution, particularly under the selective pressure of previous treatment lines [42,43]. We therefore aimed to harvest tissue samples after the last line of therapy to capture the most up-to-date mutational landscape. However, in three cases, the patient refused another biopsy, and tumor material had to be utilized, which was older than one year. Finally, it is crucial to recognize that our study was not designed as a prospective, randomized trial. Instead, it aimed to provide real-world evidence on the current role of MTB-guided precision medicine decisions for gynecologic cancer. While this approach allows for a comprehensive assessment of the current landscape, it is important to interpret the findings within the context of an observational study design.

## 4. Materials and Methods

### 4.1. Precision Oncology Program

In 2016, a biweekly interdisciplinary MTB for all oncologic indications was established as a vital component of the Precision Oncology Program at the University Hospital Munich. Its primary objective is to integrate clinical and molecular-genetic patient data into multidisciplinary discussions, ultimately providing recommendations for personalized treatment decisions. As the number of patient cases increased in 2020, the MTB doubled its meeting frequency. All patients enrolled in our Precision Oncology Program consented either to the Informed Patient Study or to SMART PRO (reference number: 21-0869; last amendment: 11/2023). Both studies were conducted at the LMU Munich and involved the storage of personal and clinical patient data in a central registry. The study protocol adheres to the Declaration of Helsinki and was approved by the ethics committee of the Faculty of Medicine, LMU Munich. 

Starting in 2017, our comprehensive review encompassed 31 cases of HPV-related gynecologic malignancies. All included patients met the following criteria: (1) histologically confirmed diagnosis of CC, vaginal cancer or vulvar cancer, (2) an Eastern Cooperative Oncology Group (ECOG) performance status of 0 or 1 and (3) interest in participating in clinical trials and/or pursuing off-label tumor therapies. 

### 4.2. Tissue and Sequencing Results

To ensure that the tissue samples accurately represented the current stage of their disease, the following criteria were established: (1) tumor samples should not be older than two years and (2) they were ideally obtained after the completion of the last standard therapy line, as previous treatments can impact tumor biology and the development of drug resistance [42,58]. None of the tumor specimens analyzed by NGS was older than twenty-four months at the time of analysis. Median interval between the initiation of molecular diagnostics and the completion of NGS analysis or the presentation of the case at the MTB was 19 days (range: 8 to 44) and 31 days (range: 15 to 67), respectively.

### 4.3. Molecular-Genetic Analysis

Molecular-genetic profiling was carried out at the Institute of Pathology, LMU Munich. To identify tumor regions rich in cancer cells, formalin-fixed paraffin-embedded (FFPE) tissue sections were meticulously examined using hematoxylin–eosin (H&E) staining. Corresponding sections were then utilized for nucleic acid extraction employing GeneRead (DNA) and RNeasy FFPE kits (RNA) (Qiagen, Hilden, Germany). Employed CGP assays testing for the indicated alterations including single- and multinucleotide variants (SNVs, MNVs), structural variants like small insertions (ins), deletions (del), indels, duplications (dup), inversions (inv) as well as copy number variations (CNVs) and RNA fusions are presented in Table 1.

Library generation for NGS involved the utilization of AmpliSeq Library Plus, AmpliSeq CD, AmpliSeq cDNA synthesis-index, AmpliSeq Equalizer and AmpliSeq for Illumina Comprehensive Panel v3 (Illumina, San Diego, CA, USA), as well as Ion AmpliSeq Library, Ion Library Equalizer, IonXpress Barcode Adapter kits and Ion Chip kits (540 and 550) (Thermo Fisher, Waltham, MA, USA) following the respective user manuals. Library sequencing was performed using an Ion Torrent GeneStudio S5 Prime (Thermo Fisher, Waltham, MA, USA) or Illumina 500/550 Next Seq (Illumina, San Diego, CA, USA) (Table 1). NGS results were analyzed using the Ion Reporter System (Thermo Fisher, Waltham, MA, USA) or Local Run Manager (Illumina, San Diego, CA, USA). Variant annotation for samples passing the quality control was performed based on VCF files using wAnnovar (https://wannovar.wglab.org/, last access date 12 January 2023). Variant interpretation was conducted utilizing the Clinical Variants ClinVar, National Institute of Health (NIH), Bethesda, MD, USA; https://www.ncbi.nlm.nih.gov/clinvar/, last access date 12 January 2023), BRCA exchange (https://brcaexchange.org/, last access date 12 January 2023), Genome Aggregation Data (gnomAD; Eli and Edythe L. Broad Institute, Boston, MA, USA; https://gnomad.broadinstitute.org/, last access date 12 January 2023), Catalogue of Somatic Mutations in Cancer (COSMIC) (Sanger Institute, Cambridge, UK; https://cancer.sanger.ac.uk/cosmic, last access date 12 January 2023) and cBioportal (Memorial Sloan Kettering Institute, New York, NY, USA; https://www.cbioportal.org/, last access date 12 January 2023) databases. Relevant variants were filtered using an in-house-developed Python (v.3.2) script called PathInfony. Variants were inspected manually with Integrative Genomics Viewer (IGV, Broad Institute, Cambridge, MA, USA) and, if required, annotation was adapted according to Human Genome Variation Society (HGVS) nomenclature. Reporting included pathogenic (ClinVar, 5) and likely pathogenic mutations (4) and variants of unknown significance (VUS, 3) with a minimum allele frequency of 3%. MTB case discussions were based on a detailed pathological report of the NGS results, along with histomorphological and immunohistochemical data (e.g., programmed death ligand 1 (PD-L1) as well as hormone receptor and human epidermal growth factor receptor 2 (Her2)).

### 4.4. Study Procedure

**Figure 10:**
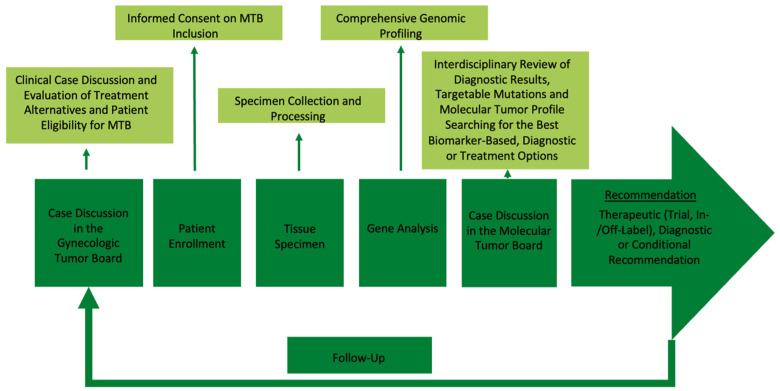
Workflow for the implementation of a molecular tumor board in clinical routine.

Figure 10 illustrates the implementation of MTB case discussions in clinical practice. Initially, all 31 patients included in this study underwent discussions at the interdisciplinary Gynecologic Tumor Board (gTB) of the Department of Obstetrics and Gynecology, University Hospital, LMU Munich. If the gTB anticipated a lack of subsequent standard therapeutic procedures, the case was referred to the MTB and molecular profiling of patient tumor samples was initiated. The resulting reports were then submitted to the MTB, which comprised a multidisciplinary team of gynecological oncologists, medical oncologists, genetic counselors, molecular biologists and bioinformaticians in accordance with the requirements of the Centers for Personalized Medicine (CPM). Subsequently, the MTB thoroughly researched current literature and databases such as PubMED, ClinVar, Varsome, OncoKB, CIViC and clinicaltrials.gov to assess the targetability of identified mutations. It furthermore considered the prevalence of these mutations in the specific patient population and examined the potential oncogenic pathways affected by the genetic alterations. This analysis aimed to identify primarily biomarker-guided, off-label or investigational drugs that could counteract the signaling defect caused by the mutation.

Based on these discussions, the MTB generated therapeutic options, accompanied by information on the level of evidence for each molecular target. The European Society for Medical Oncology (ESMO) Scale for Clinical Actionability of Molecular Targets (ESCAT) was used to assess the level of evidence supporting the recommended treatment options [59]. Finally, the MTB resulted in treatment recommendations based on the joint expertise and available evidence.

### 4.5. Software

For data analysis, Microsoft® Excel, version 16.79.1 (Microsoft, Redmont, WA, USA) was used. Illustrations were created in Microsoft® PowerPoint, version 16.81 (Microsoft, Redmont, WA, USA) and GraphPad Prism 10 for macOS (GraphPad Software Inc., Boston, MA, USA). The text was written using Microsoft® Word, version 16.79.1 (Microsoft, Redmont, WA, USA).

## 5. Conclusions

MTBs extend treatment options beyond standard of care by precision medicine approaches in patients with relapsed HPV-related gynecologic malignancies. Within our real-world data for cervical, vulvar or vaginal cancer, we could identify a considerable rate of 48% for tumors with an actionable mutation. In context of the limited treatment options of these tumor entities in the metastatic setting, these data are promising and an important perspective for the often young patients suffering from symptomatic disease. The trade-off between biomarker-guided and -agnostic targeted therapies as well as the optimal timing of individual case presentation to the MTB remains a challenge to fully realize the potential of precision medicine and requires structured and standardized processes in gynecologic cancer centers.

## Figures and Tables

**Table 1:** Overview of the molecular pathologic diagnostic panels employed.

Assay/Panel	DNA Alterations (Gene Number)	RNA/Gene Fusions (Gene Number)	CNV (Gene Number)	TMB	MSI	Sequencing Technology/Provider	Cases (N)
Oncomine Focus	52	23	19	-	-	IonTorrent, Thermo Fisher, Waltham, MA, USA	4
Oncomine comprehensive v3	161	51	43	-	-	IonTorrent, Thermo Fisher, Waltham, MA, USA	3
AmpliSeq for Illumina Comprehensive Panel v3	161	51	43	-	-	Illumina, San Diego, CA, USA	4
Oncomine tumor mutation load	409	-	-	Yes	-	IonTorrent, Thermo Fisher, Waltham, MA, USA	5
Oncomine comprehensive + Tumor mutation load	161	51	43	Yes	-	IonTorrent, Thermo Fisher, Waltham, MA, USA; Illumina, San Diego, CA, USA	9
Oncomine comprehensive plus	391	51		Yes	Yes	IonTorrent, Thermo Fisher, Waltham, MA, USA	7
TrueSightOncology (TSO) 500	523	55	59	Yes	Yes	Illumina, San Diego, CA, USA	5

## Data Availability

The raw data supporting the conclusions of this article will be made available by the authors on request.

## Supplementary Materials

The following supporting information can be downloaded at: https://www.mdpi.com/article/10.3390/ijms25042345/s1.
